# Low body mass index is associated with diminished plasma cytokines and chemokines in both active and latent tuberculosis

**DOI:** 10.3389/fnut.2023.1194682

**Published:** 2023-05-30

**Authors:** Nathella Pavan Kumar, Arul P. Nancy, Kadar Moideen, Pradeep A. Menon, Vaithilingam V. Banurekha, Dina Nair, Sujatha Nott, Subash Babu

**Affiliations:** ^1^ICMR-National Institute for Research in Tuberculosis, Chennai, India; ^2^NIAID – International Center for Excellence in Research, Chennai, India; ^3^Infectious Diseases, Dignity Health, Chandler, AZ, United States; ^4^LPD, NIAID, NIH, Rockville, MD, United States

**Keywords:** tuberculosis, body mass index, cytokines, chemokines, latent tuberculosis

## Abstract

**Introduction:**

Low body mass index (BMI) is a major risk factor for tuberculosis (PTB). Low BMI can impair the immune system and thus might affect TB incidence.

**Methods:**

We examined the plasma levels of Type 1, Type 17, pro-inflammatory, Type 2 and regulatory cytokines and CC and CXC chemokines in PTB and latent TB (LTB) individuals with low BMI (LBMI) or normal BMI (NBMI).

**Results:**

Our data show that PTB is associated with significantly lower levels of IFN*γ*, TNF*α*, IL-2, IL-17A, IL-6, IL-12, IL-4 and IL-5 cytokines but significantly higher levels of IL-10, TGF*β* and GM-CSF in LBMI compared to NBMI. Similarly, PTB is also associated with significantly lower levels of CCL2, CCL3, CCL11, CXCL1, CXCL9 and CXCL10 chemokines in LBMI compared to NBMI. Our data reveals that LTB is associated with significantly lower levels of IFN*γ*, TNF*α*, IL-2, IL1*β*, IL-12, IL-13 cytokines but significantly higher levels of IL-10, TGF*β*, IL-4 and IL-22 in LBMI compared to NBMI. Similarly, LTB is also associated with significantly lower levels of CCL2, CXCL1, CXCL9 and CXCL10 and significantly higher levels of CCL1, CCL3, and CCL4 in LBMI compared to NBMI.

**Conclusion:**

Thus, LBMI has a major impact on the cytokine and chemokine milieu of both PTB and LTB and might predispose to the increased risk of tuberculosis by this immunomodulatory effect.

## Introduction

Low body mass index (BMI) is the leading risk factor for tuberculosis (TB) with a worldwide population attributable fraction of 19% in 2018, which is higher than that of HIV and diabetes mellitus (1). Various studies have shown that low BMI is associated with increased risk of TB (2). Low BMI is also associated with increased severity of disease (in the form of cavitation and lung involvement) and increased risk of unfavorable TB treatment outcomes (including treatment failure and relapse) (1, 3). Low BMI is also associated with heightened mortality in different studies of pulmonary TB (PTB) (4, 5). Therefore, low BMI as a comorbidity plays a very significant role in PTB pathogenesis and progression. The effect of low BMI on the incidence of latent *Mycobacterium tuberculosis* infection (LTB) is less well characterized. A recent meta-analysis of the effect of low BMI on LTB incidence did not show any significant association (6). Our study on the proximate risk factors of TB in LTBI individuals also did not reveal any association with BMI (7). However, low BMI is known to exert major immunological effects on PTB as well as LTB.

A wealth of data suggest that low BMI is associated with impairment of innate and adaptive immune responses responsible for the control of TB infection and might affect responses to live vaccines, such as BCG but this is based mostly on studies in animal models (1, 8). Thus, low BMI is known to be associated with diminished antigen-specific cytokine responses (9), impaired antigen-specific chemokine responses (10), perturbed T cell, B cell, dendritic cell and monocyte subsets (11) and diminished anti-microbial peptide responses in LTB (12). Similarly, low BMI is associated with diminished pro-inflammatory cytokines (13) in PTB and a minimal impact on systemic cytokine and chemokine responses in tuberculous lymphadenitis (14).

Therefore, more studies are needed to explore the immunological underpinnings of the interaction between low BMI and PTB/LTB. To fill this knowledge gap, we examined the cytokine and chemokine milieu in low BMI individuals with either PTB or LTB and demonstrate that low BMI is associated with diminished plasma cytokine and chemokine levels in both TB infection and disease.

## Materials and methods

### Study population

We studied a group of 253 individuals with active PTB, 127 with low BMI (hereafter LBMI) and 126 with normal BMI (hereafter NBMI) (Table 1). Individuals with PTB were diagnosed by positive AFB smear grade and solid cultures in Lowenstein–Jensen medium. All participants with PTB had no record of prior TB disease or ATT at the time of enrolment. All PTB individuals were recruited for hospitals in Chennai. In addition, we also studied a group of 176 individuals with LTB, 71 with LBMI and 105 with NBMI (Table 1). LTB individuals were screened as part of a community screening protocol in a rural village on the outskirts of Chennai. All individuals were adults of between 18 and 65 years of age and were enrolled consecutively. LTB was diagnosed on the basis of being tuberculin skin test (TST) positive and Quantiferon TB Gold in Tube test (QFT) positive, with no symptoms or signs of active TB, no history of previous TB, and normal chest radiographs. TST was performed using 2 tuberculin units of tuberculin purified protein derivative (PPD) RT 23 SSI (Serum Statens Institute). A positive skin test was defined as an induration of at least 12 mm in diameter, based on the previously determined cutoff norms for South India. Low and normal BMIs were defined on the basis of the 2013 American Heart Association/American College of Cardiology guidelines (LBMI, <18.5 kg/m^2^; and NBMI, between 18.5 and 24.9 kg/m^2^). The guidelines are the same for both males and females. HIV and diabetes mellitus were exclusion criteria for both PTB and LTB.

**Table 1:** Demographics of the study groups.

	Pulmonary TB		Latent TB	
	LBMI	NBMI	LBMI	NBMI
Number of subjects recruited	127	126	71	105
Gender (Male/Female)	108/19	86/40	35/36	48/57
Median age (Range) (in years)	39 (25–67)	50 (26–73)	32 (18–62)	31 (18–61)
BMI (Median)	16 (13.2–18.1)	22 (19.8–32)	17 (14–17.2)	21 (18.9–23)
Tuberculin skin test	Not done	Not done	Positive	Positive
Quantiferon TB gold	Negative	Negative	Positive	Positive

The values represent the geometric mean (and the 95% confidence intervals) except for age where the median (and the range) are depicted.

### Plasma cytokines and chemokines

Circulating levels of cytokines and chemokines were measured using Bio-Plex multiplex assay system (Bio-Rad, Hercules, CA). Circulating levels of IFN*γ*, IL-2, TNF*α*, IL-17A, IL-22, IL-1*β*, IL-6, IL-12, GM-CSF, IL-4, IL-5, IL-13 and IL-10 were measured using the Bio-Plex multiplex cytokine assay system (Bio-Rad, Hercules, CA). TGF*β* alone was measured using the R & D Systems Quantikine ELISA. The lowest detection limits were as follows: IFN*γ*, 4.39 pg/mL; IL-2, 29.63 pg/mL; TNF*α*, 3.24 pg/mL; IL-17A, 1.69 pg/mL; IL-22, 12.63 pg/mL; IL-1*β*, 3.96; IL-6, 7.7 pg/mL; IL-12, 4.66 pg/mL; GM-CSF, 12.22 pg/mL; IL-4, 14.61 pg/mL; IL-5, 6.63 pg/mL; IL-13, 216.2 pg/mL; IL-10, 4.65 pg/mL and TGF*β*, 7.8 pg/mL. Luminex Human Chemokines Magnetic Assay kit (R & D systems) was used to measure the chemokine levels of CCL1, CCL2, CCL3, CCL4, CCL-11, CXCL1, CXCL2, CXCL9, CXCL10, and CXCL11. The lowest detection limits were as follows: CCL1, 1.57 pg/mL; CCL2, 31.8 pg/mL; CCL3, 90.9 pg/mL; CCL-4, 133.1 pg/mL, CCL11, 21.6 pg/mL; CXCL1, 49.2 pg/mL; CXCL2, 49.2 pg/mL; CXCL9, 600.6 pg/mL CXCL10, 2.88 pg/mL and CXCL11, 21.6 pg/mL.

### Statistical analysis

Geometric means (GM) were used for measurements of central tendency. Statistically significant differences between two groups were analysed using the nonparametric Mann–Whitney U test with Holm’s correction for multiple comparisons. Correlations were calculated using Spearman rank correlation. *p* values of <0.005 were considered significant. Analyses were performed using GraphPad Prism, version 8.01 and JMP 13.0 (SAS, Cary, NC, United States).

## Results

### Low BMI is associated with diminished plasma cytokines in PTB

**Figure 1:**
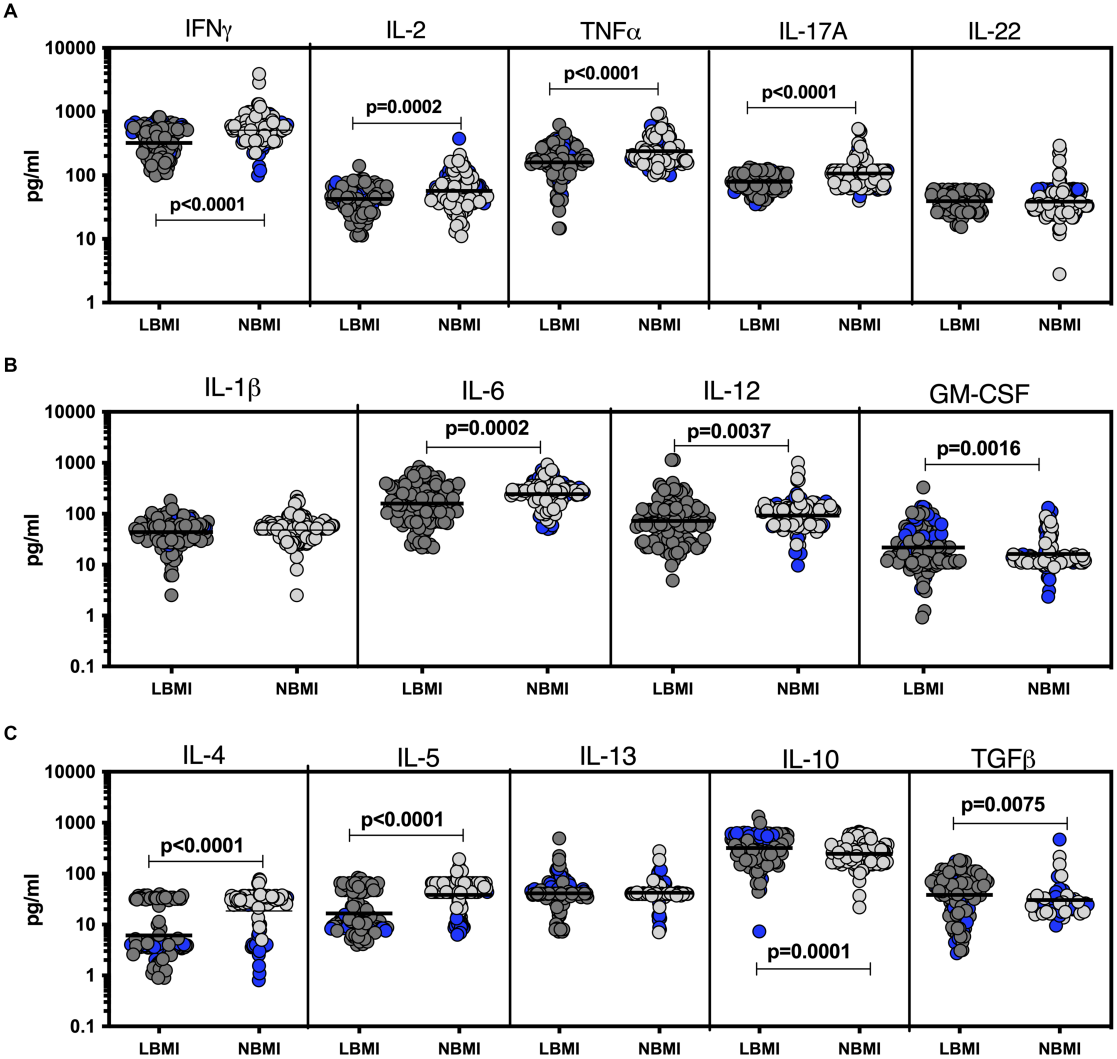
Diminished plasma cytokines in PTB patients with LBMI. The plasma levels of cytokines **(A)** Type 1 (IFN*γ*, TNF*α*, IL-2) and Type 17 (IL-17A, IL-22) **(B)** pro-inflammatory (IL-1*β*, IL-6, IL-12, GM-CSF) and **(C)** Type 2 (IL-4, IL-5, IL-13) and regulatory (IL-10 and TGF*β*) were measured in PTB individuals with LBMI (*n* = 127) and NBMI (*n* = 126). The data are represented as scatter plots with each circle representing a single individual. Female gender was coded with blue colour. *p* values were calculated using the Mann–Whitney test.

To estimate the levels of plasma cytokines in LBMI-PTB co-morbidity, we measured the levels of Type 1 (IFN*γ*, TNF*α*, IL-2), Type 17 (IL-17A, IL-22), pro-inflammatory (IL-1*β*, IL-6, IL-12, GM-CSF), Type 2 (IL-4, IL-5, IL-13) and regulatory (IL-10 and TGF*β*) cytokines in PTB individuals with LBMI (*n* = 127) or NBMI (*n* = 126). As shown in Figure 1A, the levels of IFN*γ* (GM of 323.3 pg/mL for LBMI versus 511.5 pg/mL for NBMI; *p* = 0.0001), IL-2 (GM of 42.5 pg/mL for LBMI versus 57 pg/mL for NBMI; *p* < 0.0001), TNF*α* (GM of 159.5 pg/mL for LBMI versus 238.2 pg/mL for NBMI; *p* < 0.0001) and IL-17A (GM of 78.9 pg/mL for LBMI versus 106.7 pg/mL for NBMI; *p* < 0.0001) were significantly lower in LBMI compared to NBMI individuals with PTB. Also, as shown in Figure 1B, the levels of IL-6 (GM of 159.6 pg/mL for LBMI versus 242.1 pg/mL for NBMI; *p* = 0.0002) and IL-12 (GM of 72.1 pg/mL for LBMI versus 92.5 pg/mL for NBMI; *p* = 0.0037) were significantly lower and the levels of GM-CSF (GM of 21.8 pg/mL for LBMI versus 16.2 pg/mL for NBMI; *p* = 0.0016) were significantly higher in LBMI compared to NBMI individuals in PTB. Finally, as shown in Figure 1C, the levels of IL-4 (GM of 6 pg/mL for LBMI versus 18 pg/mL for NBMI; *p* < 0.0001) and IL-5 (GM of 16.6 pg/mL for LBMI versus 38.3 pg/mL for NBMI; *p* < 0.0001) were significantly lower and the levels of IL-10 (GM of 319.2 pg/mL for LBMI versus 245.4 pg/mL for NBMI; *p* = 0.0001) and TGF*β* (GM of 38.2 pg/mL for LBMI versus 26.4 pg/mL for NBMI; *p* = 0.0075) were significantly higher in LBMI compared to NBMI individuals with PTB. Thus, low BMI is associated with mostly diminished levels of cytokines in PTB.

### Low BMI is associated with diminished plasma chemokines in PTB

**Figure 2:**
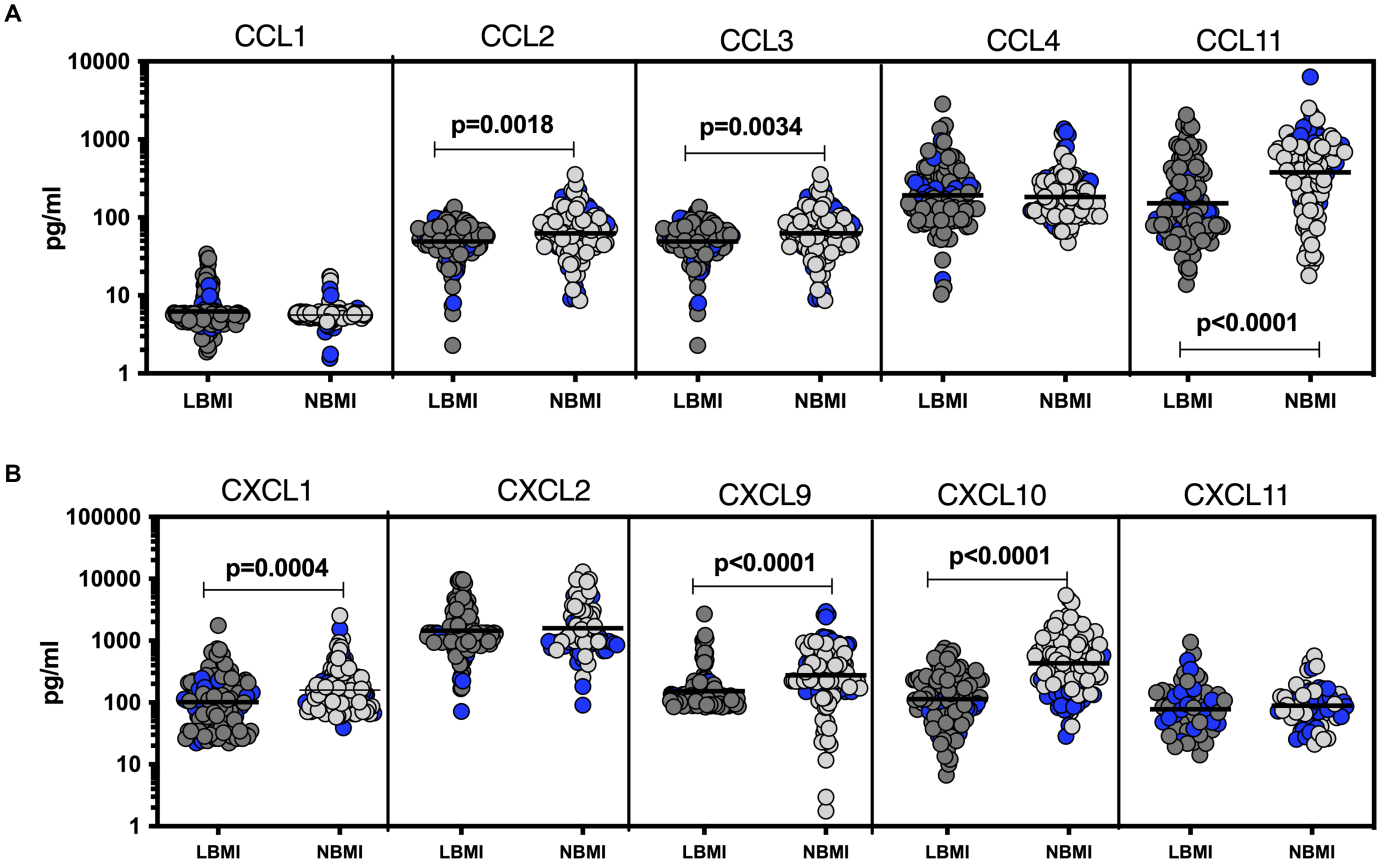
Diminished plasma chemokines in PTB patients with LBMI. The plasma levels of chemokines **(A)** CC (CCL1, CCL2, CCL3, CCL4, CCL-11) and **(B)** CXC CXCL1, CXCL2, CXCL9, CXCL10 and CXCL11 were measured in PTB individuals with LBMI (*n* = 127) and NBMI (*n* = 126). The data are represented as scatter plots with each circle representing a single individual. Female gender was coded with blue colour. *p* values were calculated using the Mann–Whitney test.

To estimate the levels of plasma chemokines in LBMI-PTB co-morbidity, we measured the levels of CC (CCL1, CCL2, CCL3, CCL4, CCL11) and CXC (CXCL1, CXCL-2, CXCL9, CXCL10, CXCL11) chemokines in PTB individuals with LBMI (*n* = 127) or NBMI (*n* = 126). As shown in Figure 2A, the levels of CCL2 (GM of 49.2 pg/mL for LBMI versus 62.5 pg/mL for NBMI; *p* = 0.0018), CCL3 (GM of 43.5 pg/mL for LBMI versus 53.6 pg/mL for NBMI; *p* = 0.00374) and CCL11 (GM of 152.1 pg/mL for LBMI versus 379.2 pg/mL for NBMI; *p* < 0.0001) were significantly lower in LBMI compared to NBMI individuals with PTB. Similarly, as shown in Figure 2B, the levels of CXCL1 (GM of 101.5 pg/mL for LBMI versus 158.6 pg/mL for NBMI; *p* = 0.0004), CXCL9 (GM of 152.4 pg/mL for LBMI versus 274.5 pg/mL for NBMI; *p* < 0.0001) and CXCL10 (GM of 113.4 pg/mL for LBMI versus 230.3 pg/mL for NBMI; *p* = 0.0001) were significantly lower in LBMI compared to NBMI individuals in PTB. Thus, low BMI is associated with mostly diminished levels of chemokines in PTB.

### Low BMI is associated with altered plasma cytokines in LTB

**Figure 3:**
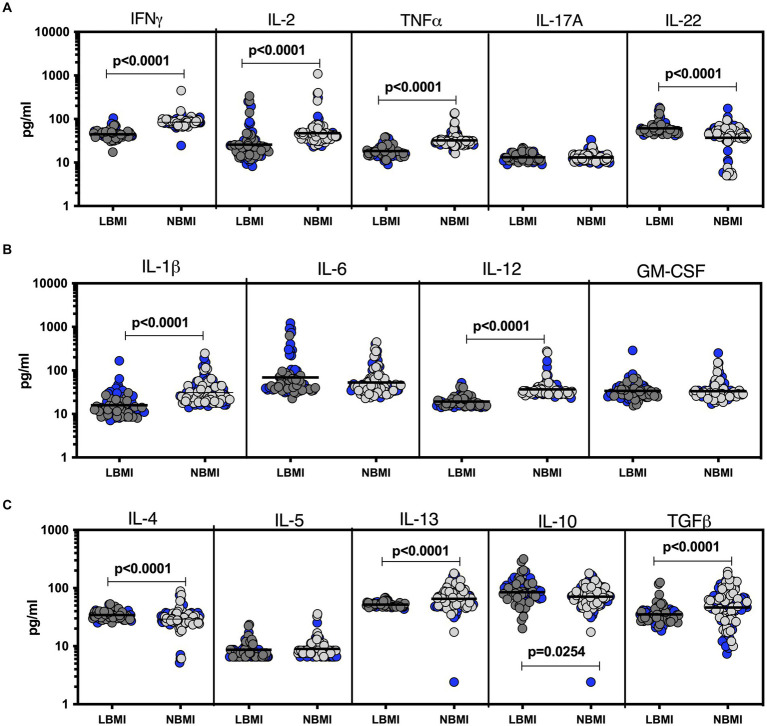
Altered plasma cytokines in LTB individuals with LBMI. The plasma levels of cytokines **(A)** Type 1 (IFN*γ*, TNF*α*, IL-2) and Type 17 (IL-17A, IL-22) **(B)** pro-inflammatory (IL-1*β*, IL-6, IL-12, GM-CSF) and **(C)** Type 2 (IL-4, IL-5, IL-13) and regulatory (IL-10 and TGF*β*) were measured in PTB individuals with LBMI (*n* = 71) and NBMI (n = 105). The data are represented as scatter plots with each circle representing a single individual. Female gender was coded with blue colour. *p* values were calculated using the Mann–Whitney test.

To estimate the levels of plasma cytokines in LBMI-LTB co-morbidity, we measured the levels of Type 1 (IFN*γ*, TNF*α*, IL-2), Type 17 (IL-17A, IL-22), pro-inflammatory (IL-1*β*, IL-6, IL-12, GM-CSF), Type 2 (IL-4, IL-5, IL-13) and regulatory (IL-10 and TGF*β*) cytokines in LTB individuals with LBMI (*n* = 71) or NBMI (*n* = 105). As shown in Figure 3A, the levels of IFN*γ* (GM of 44.7 pg/mL for LBMI versus 84.5 pg/mL for NBMI; *p* < 0.0001), IL-2 (GM of 25.6 pg/mL for LBMI versus 47.1 pg/mL for NBMI; *p* < 0.0001) and TNF*α* (GM of 18.4 pg/mL for LBMI versus 32.3 pg/mL for NBMI; *p* < 0.0001) were significantly lower and the levels of IL-22 (GM of 61.1 pg/mL for LBMI versus 37 pg/mL for NBMI; *p* < 0.0001) significantly higher in LBMI compared to NBMI individuals with LTB. Also, as shown in Figure 3B, the levels of IL-1*β* (GM of 15.8 pg/mL for LBMI versus 31.1 pg/mL for NBMI; *p* < 0.0001) and IL-12 (GM of 19.3 pg/mL for LBMI versus 36.6 pg/mL for NBMI; *p* < 0.0001) were significantly lower in LBMI compared to NBMI individuals in LTB. Finally, as shown in Figure 3C, the levels of IL-10 (GM of 19.3 pg/mL for LBMI versus 36.6 pg/mL for NBMI; *p* < 0.0001) and IL-4 (GM of 34.3 pg/mL for LBMI versus 29.3 pg/mL for NBMI; *p* < 0.0001) were significantly higher in LBMI compared to NBMI individuals with LTB. Thus, low BMI is associated with altered levels of cytokines in LTB.

### Low BMI is associated with altered plasma chemokines in LTB

**Figure 4:**
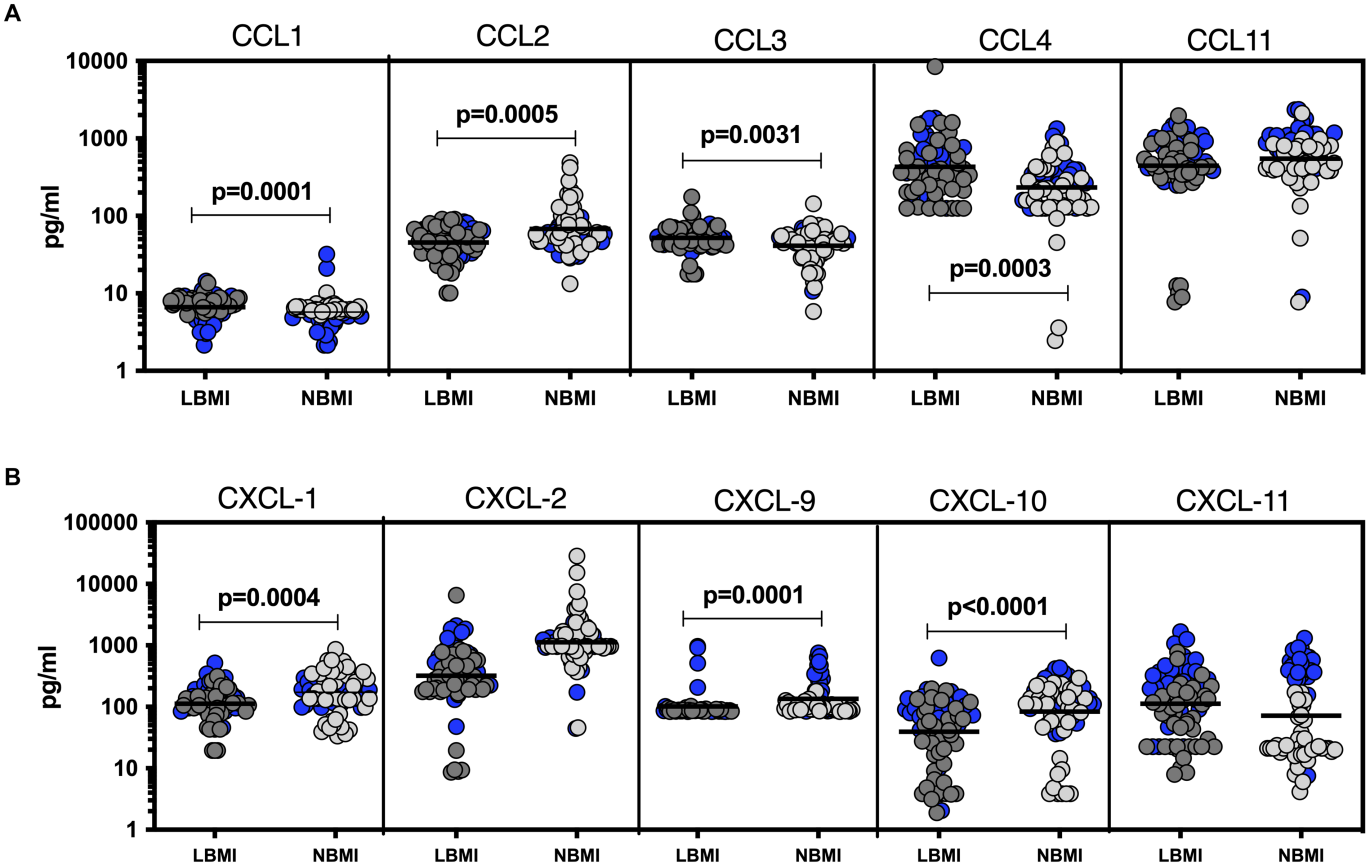
Altered plasma chemokines in LTB individuals with LBMI. The plasma levels of chemokines **(A)** CC (CCL1, CCL2, CCL3, CCL4, CCL11) and **(B)** CXC CXCL1, CXCL2, CXCL9, CXCL10 and CXCL11 were measured in PTB individuals with LBMI (*n* = 71) and NBMI (*n* = 105). The data are represented as scatter plots with each circle representing a single individual. Female gender was coded with blue colour. *p* values were calculated using the Mann–Whitney test.

To estimate the levels of plasma chemokines in LBMI-LTB co-morbidity, we measured the levels of CC (CCL1, CCL2, CCL3, CCL4, CCL11) and CXC (CXCL1, CXCL-2, CXCL9, CXCL10, CXCL11) chemokines in LTB individuals with LBMI (*n* = 127) or NBMI (*n* = 126). As shown in Figure 4A, the levels of CCL2 (GM of 45.3 pg/mL for LBMI versus 67.8 pg/mL for NBMI; *p* = 0.0005) were significantly lower and the levels of CCL1 (GM of 6.7 pg/mL for LBMI versus 5.2 pg/mL for NBMI; *p* = 0.0001), CCL3 (GM of 51.8 pg/mL for LBMI versus 41 pg/mL for NBMI; *p* = 0.0031) and CCL4 (GM of 431.8 pg/mL for LBMI versus 234.3 pg/mL for NBMI; *p* = 0.0013) were significantly higher in LBMI compared to NBMI individuals with LTB. Also, as shown in Figure 4B, the levels of CXCL1 (GM of 112.4 pg/mL for LBMI versus 172.8 pg/mL for NBMI; *p* = 0.0004), CXCL9 (GM of 101.7 pg/mL for LBMI versus 135.1 pg/mL for NBMI; *p* = 0.0001) and CXCL10 (GM of 39.5 pg/mL for LBMI versus 83.4 pg/mL for NBMI; *p* < 0.0001) were significantly lower in LBMI compared to NBMI individuals in LTB. Thus, low BMI is associated with altered levels of chemokines in LTB.

### Low BMI is associated with altered plasma cytokines and chemokines in PTB and LTB

To estimate the systemic levels of plasma cytokines and chemokines in PTB-LBMI and LTB-LBMI, we analysed the levels of type 1, type 2, type 17 and other pro-inflammatory cytokines and CC and CXC chemokines in PTB individuals with LBMI (*n* = 127) and LTB individuals with LBMI (*n* = 71). As shown in the Table 2, cytokines such as IFN*γ*, TNF*α*, IL-17A, IL-1*β*, IL-6, IL-12, IL-5, IL-10 were significantly higher in PTB-LBMI compared to LTB-LBMI. However, cytokines such as IL-22, GM-CSF, IL-4 and IL-13 were significantly lower in PTB-LBMI compared to LTB-LBMI. Similarly, chemokines such as CXCL2, CXCL9 and CXCL10 were significantly higher in PTB-LBMI compared to LTB-LBMI, while conversely chemokines such as CCL4 and CCL11 were significantly lower in PTB-LBMI compared to LTB-LBMI Thus, low BMI is associated with altered levels of cytokine and chemokines in both tuberculosis disease and infection.

**Table 2:** Altered plasma cytokines and chemokines in PTB and LTB individuals with LBMI.

Parameters	PTB-LBMI	LTB-LBMI	*p* value
	GeoMean	GeoMean	
IFN*γ*	323.3	44.72	<0.0001
IL-2	42.56	42.56	0.9993
TNF*α*	159.5	18.43	<0.0001
IL-17A	78.93	13.22	<0.0001
IL-22	39.09	61.15	<0.0001
IL-1*β*	43.65	15.88	<0.0001
IL-6	159.6	68.99	<0.0001
IL-12	72.13	19.31	<0.0001
GM-CSF	21.87	33.89	<0.0001
IL-4	6.095	34.37	<0.0001
IL-5	16.63	8.66	<0.0001
IL-13	41.31	51.84	<0.0001
IL-10	319.2	84.83	<0.0001
TGF*β*	38.24	35.21	0.0061
CCL1	6.206	6.634	0.0022
CCL2	49.23	45.39	0.1159
CCL3	55.22	51.87	0.8805
CCL4	192.7	431.8	<0.0001
CCL11	152.1	446.2	<0.0001
CXCL1	101.5	112.4	0.3406
CXCL2	1,451	322.2	<0.0001
CXCL9	152.4	101.7	<0.0001
CXCL10	113.4	39.5	<0.0001
CXCL11	78	112.6	0.0629

The values represent the geometric mean (and the 95% confidence intervals) are depicted. *p* values were calculated using the Mann–Whitney test.

### Relationship between plasma cytokine/chemokine levels and BMI in PTB

**Figure 5:**
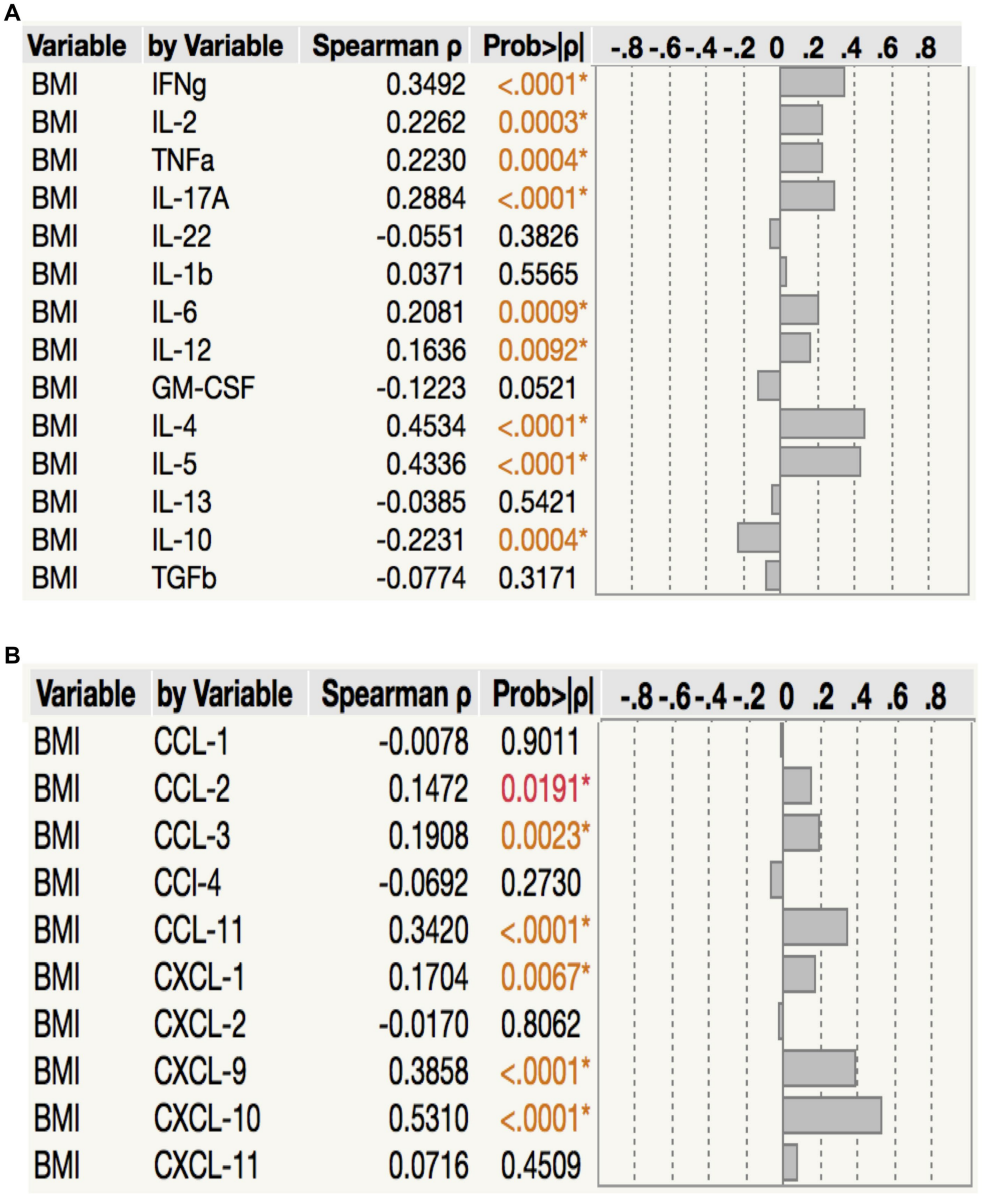
Relationship between cytokines, chemokines and BMI. in PTB **(A)** The relationship between the plasma levels of cytokines and BMI in all PTB individuals with LBMI and NBMI. **(B)** The relationship between the plasma levels of chemokines and BMI in all PTB individuals with LBMI and NBMI. *p* values were calculated using the Spearman Rank Correlation.

To assess the relationship between plasma cytokine levels and BMI in PTB individuals, we performed Spearman rank correlation analysis between the two parameters. As shown in Figure 5A, correlation analysis revealed that BMI was positively associated with IFN*γ* (*p* < 0.0001, *r* = 0.3492), IL-2 (*p* = 0.0003, *r* = 0.2262), TNF*α* (*p* = 0.0004, *r* = 0.2230), IL-17A (*p* < 0.0001, *r* = 0.2884), IL-6 (*p* = 0.0009, *r* = 0.2081), IL-12 (*p* = 0.0092, *r* = 0.1636), IL-4 (*p* < 0.0001, *r* = 0.4534) and IL-5 (*p* < 0.0001, *r* = 0.4336) but negatively associated with IL-10 (*p* = 0.0004, *r* = −0.2231) in PTB. Similarly, as shown in Figure 5B, correlation analysis revealed that BMI was positively associated with CCL2 (*p* = 0.0191, *r* = 0.1472), CCL3 (*p* = 0.0023, *r* = 0.1908), CCL11 (*p* < 0.0001, *r* = 0.3420), CXCL1 (*p* = 0.0067, *r* = 0.1704), CXCL9 (*p* < 0.0001, *r* = 0.3858) and CXCL10 (*p* < 0.0001, *r* = 0.5310) in PTB. Thus, plasma cytokines and chemokines exhibit a significant albeit very modest positive association with BMI in PTB.

### Relationship between plasma cytokine/chemokine levels and BMI in LTB

**Figure 6:**
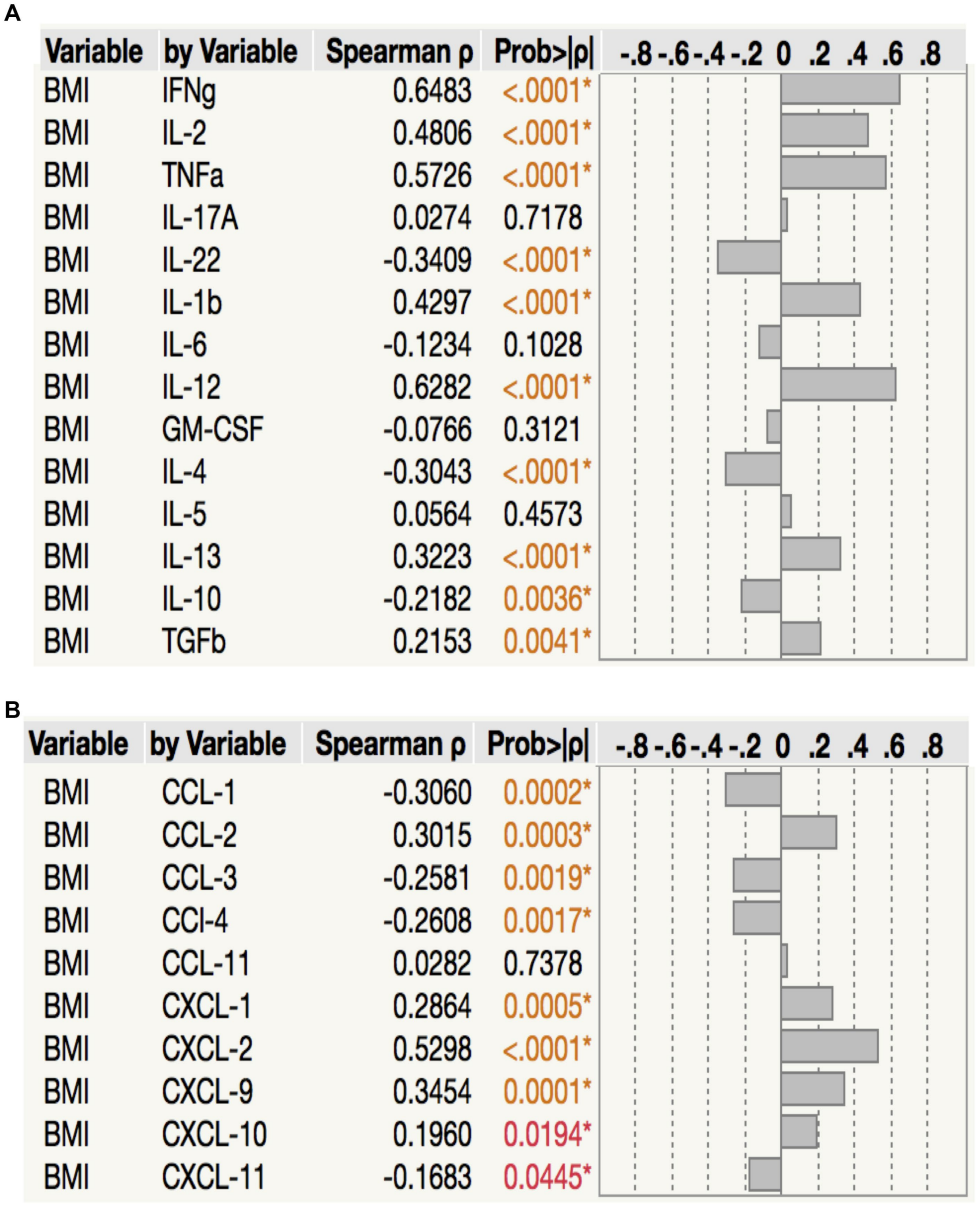
Relationship between cytokines, chemokines and BMI in LTB. **(A)** The relationship between the plasma levels of cytokines and BMI in all LTB individuals with LBMI and NBMI. **(B)** The relationship between the plasma levels of chemokines and BMI in all LTB individuals with LBMI and NBMI. *p* values were calculated using the Spearman Rank Correlation.

To assess the relationship between plasma chemokine levels and BMI in LTB individuals, we performed Spearman rank correlation analysis between the two parameters. As shown in Figure 6A, correlation analysis revealed that BMI was positively associated with IFN*γ* (*p* < 0.0001, *r* = 0.6483), IL-2 (*p* < 0.0001, *r* = 0.4806) TNF*α* (*p* < 0.0001, *r* = 0.5726), IL-1*β* (*p* < 0.0001, *r* = 0.4297), IL-12 (*p* < 0.0001, *r* = 0.6282), IL-13 (*p* < 0.0003, *r* = 0.3223) and TGF*β* (*p* = 0.0041, *r* = 0.2153) but negatively associated with IL-22 (*p* < 0.0001, *r* = −0.3409), IL-4 (*p* < 0.0001, *r* = −0.3043) and IL-10 (*p* = 0.0036, *r* = −0.2182) in LTB. As shown in Figure 6B, correlation analysis revealed that BMI was positively associated with CCL2 (*p* = 0.0001, *r* = 0.3454), CXCL1 (*p* = 0.0005, r = 0.2864), CXCL2 (*p* < 0.0001, *r* = 0.5298), CXCL9 (*p* = 0.0001, *r* = 0.3454) and CXCL10 (*p* = 0.0194, *r* = 0.1960) but negatively associated with CCL1 (*p* = 0.0002, *r* = −0.3060), CCL3 (*p* = 0.0019, *r* = −0.2581), CCL4 (*p* = 0.0017, *r* = 0.2608) and CXCL11 (*p* = 0.0445, *r* = −0.1683) in LTB. Thus, plasma cytokines and chemokines exhibit a mixed association with BMI in LTB.

## Discussion

Low BMI can influence innate and adaptive immune responses in TB by a variety of mechanisms (15). Plasma cytokines and chemokines are both correlates of protective immunity in latent TB, diagnostic markers of active TB and associated coinfections and comorbidities, markers of disease severity and bacterial burden in active TB (16–19). The effect of low BMI on cytokine and chemokine responses in PTB and LTB has not been explored fully. Our comprehensive analysis of plasma cytokines and chemokines clearly indicates that low BMI is associated with diminished cytokine/chemokine responses in active TB and modulated cytokine/chemokine responses in latent TB. Type 1 and Type 17 cytokines are known to be major players of resistance to active TB in both animal models and human disease (20, 21). Similarly, a diverse set of pro-inflammatory cytokines including IL-1*β*, IL-6, IL-12 and GM-CSF are known to exert protective immunity to *M. tuberculosis* in animal models (16, 22, 23). In contrast, Type 2 cytokines, including IL-4, IL-5 and IL-13 and regulatory cytokines, including IL-10 and TGF*β* are known to promote susceptibility to *M. tuberculosis* in animal models (21, 22).

Our data on PTB and LTB individuals with low BMI clearly shows a major effect of BMI on these panel of cytokines. First, low BMI with PTB is characterized by diminished levels of IFN*γ*, TNF*α*, IL-2, IL-17, IL-6 and IL-12, indicating a lack of protective immunity in these individuals. Second, Type 2 cytokines involved are known to induce susceptibility to TB infection and disease (24) However, our data suggest that even in the presence of TB disease, IL-4 and IL-5 levels are low, possibly due to the predominant influence of malnutrition in suppressing Th2 responses. Third, IL-10, TGF*β* and GM-CSF are present at higher levels in PTB individuals with low BMI, which in the case of IL-10 and TGF*β* are expected since they are known to be associated with increased levels in low BMI in general. Fourth, low BMI with LTB is also associated with diminished levels of IFN*γ*, TNF*α*, IL-2, IL-1*β*, and IL-12, again indicating the importance of these cytokines in protective immunity to LTB as well. Fifth, similar to PTB individuals, low BMI with LTB is also associated with elevated levels of IL-10 and TGF*β* but in addition, also IL-4 and IL-22. While IL-10 and TGF*β* are associated with immune regulation (25), IL-4 and IL-22 possibly are involved in systemic wound healing and repair and hence, present at elevated levels.

Chemokines are important players in LTB, including in the formation and maintenance of dormant granulomas and in the recruitment of cells to form the granuloma (26, 27). Chemokines are known to be modulated in PTB and LTB (28) but the impact of BMI is not known. Our data on PTB and LTB individuals with low BMI also clearly major effects of BMI on our panel of chemokines examined. First, low BMI with PTB is associated with diminished levels of CC chemokines – CCL2, CCL3 and CCL11. Second, low BMI with PTB is also associated with diminished levels of CXC chemokines – CXCL1, CXCL9 and CXCL10. Third, similar to low BMI with PTB, low BMI with LTB is also associated with lower levels of CCL2, CXCL1, CXCL9 and CXCL10. Fourth, in contrast to low BMI with PTB, low BMI with LTB is associated with higher levels of CCL1, CCL3 and CCL4. Thus, while for the most part, low BMI is associated with decreased levels of chemokines, a few chemokines are elevated (only in LTB) in PTB and LTB. Since, the chemokine panel examined in this study are all associated with protective immunity to PTB and LTB in animal models (16), our data suggest that low BMI might compromise immunity to *M. tuberculosis* in both active and latent TB.

Finally, our study also performs correlation analysis of the whole panel of cytokines and chemokines with BMI in both PTB and LTB individuals. Our data clearly reveals a mostly negative relationship between Type 1/Type 17 and other pro-inflammatory cytokines, as well as pro-inflammatory chemokines in both PTB and LTB. Our data also reveal a mostly positive association of regulatory cytokines (and to a lesser extent Type 2 cytokines) and related chemokines with BMI. Our study suffers from the limitations of being cross-sectional and therefore not proving causality, of not having post-treatment data on PTB individuals and not accounting for other confounding variables. Another limitation is that there is no equal distribution between the genders in PTB groups. Our study strengths include large sample size and exclusion of HIV and diabetes mellitus, which are the known confounders of the immune response in PTB and LTB individuals. Our other strength was the inclusion of a large panel of innate and adaptive immune parameters to be analysed.

Thus, our study offers new insights in the immunological underpinnings of the low BMI – tuberculosis co-morbidity in both TB disease and infection. Our study also offers a plausible biological mechanism for the increased susceptibility of low BMI individuals to TB disease and perhaps infection. Further studies on other immune parameters should provide more valuable clues in underlying the mechanism of this deleterious interaction.

## Data availability statement

The original contributions presented in the study are included in the article/supplementary material, further inquiries can be directed to the corresponding author.

## Ethics statement

The studies involving human participants were reviewed and approved by ICMR-NIRT Ethics committee. The patients/participants provided their written informed consent to participate in this study.

## Author contributions

SB and NK: designed the study and wrote the manuscript. NK, KM, and AN: conducted experiments. NK and KM: acquired data and analysed data. SB: funding acquisition. PM, VB, DN, and SN: project administration. All authors contributed to the article and approved the submitted version.

## Funding

This work was funded in part by the Division of Intramural Research, NIAID, NIH.

## Conflict of interest

The authors declare that the research was conducted in the absence of any commercial or financial relationships that could be construed as a potential conflict of interest.

## Publisher’s note

All claims expressed in this article are solely those of the authors and do not necessarily represent those of their affiliated organizations, or those of the publisher, the editors and the reviewers. Any product that may be evaluated in this article, or claim that may be made by its manufacturer, is not guaranteed or endorsed by the publisher.
